# Supplementary figures and images for: Development of an RNA Nanostructure for Effective Botrytis cinerea Control through Spray-Induced Gene Silencing without an Extra Nanocarrier
Source: J Fungi (Basel). 2024 Jul 14;10(7):483. doi: 10.3390/jof10070483 (PMC11277573; doi:10.3390/jof10070483)

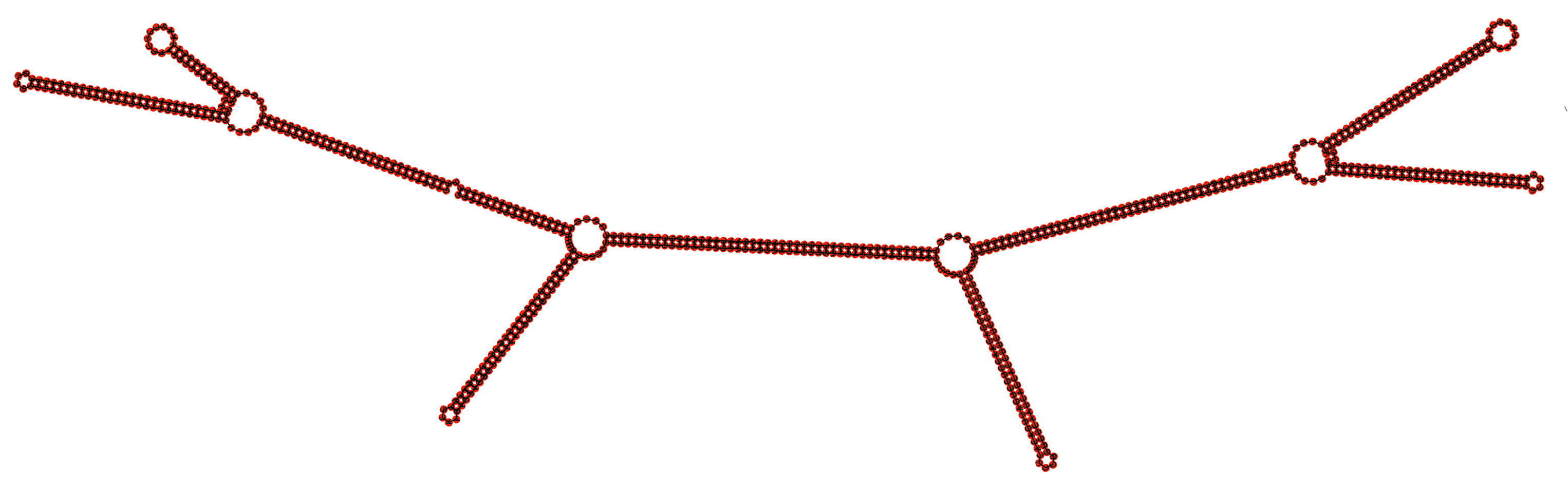

Supplement: Supplementary file 1 [file jof-10-00483-s001.zip › Figure S1.jpg]
